# Supplementary material for: Resveratrol, EGCG and Vitamins Modulate Activated T Lymphocytes
Source: Molecules. 2021 Sep 15;26(18):5600. doi: 10.3390/molecules26185600 (PMC8470394; doi:10.3390/molecules26185600)
Supplement: Supplementary file 1 [file molecules-26-05600-s001.zip › molecules-1323271-supplementary.pdf]

molecules

MDPI

RESVERATROL, EGCG AND VITAMINS MODULATE ACTIVATED T LYMPHOCYTES

Figure S1: Cells were negatively selected for CD3+, CD4+ and CD8+ lymphocytes and stained for CD4 and CD8. Numbers in the quadrants indicate the percentage of positive cells

Figure S1a: Staining of CD3+-selected PBMCs with anti-CD4 and anti-CD8.

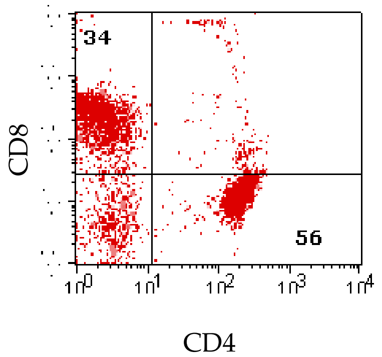

Figure S1b: Staining of CD4+-selected PBMC with anti-CD4 and anti-CD8

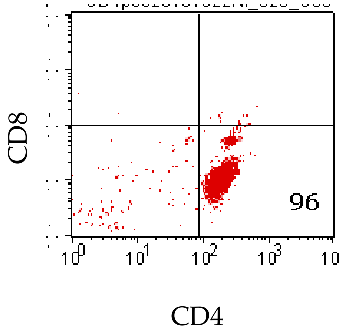

Figure S1c: Staining of CD8+-selected PBMC with anti-CD4 and anti-CD8.

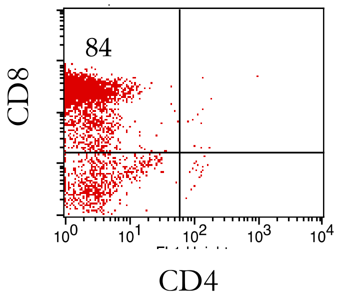

Figure S2: Anti-CD3/CD28 activated cells were cultured for 5 days in the presence of the indicated substances and stained for CD4 and CD8 determinants. Numbers in the quadrants indicate the percentage of positive cells. Cells cultured in the absence of substances had a staining profile similar to 'Res'-cells.

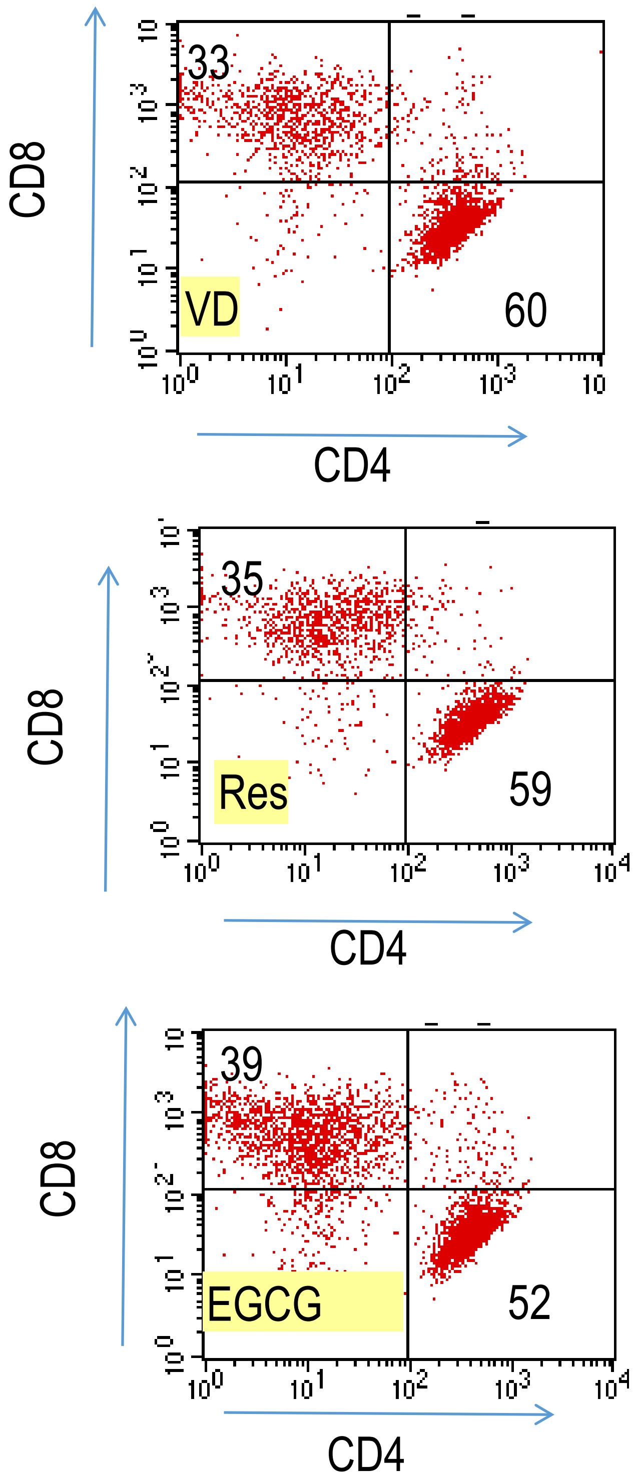

Table S1a: Production of interleukins and cytokines by CD4⁺ and CD8⁺ subsets

CD4⁺ or CD8⁺ selected PBMC subsets were activated with anti-CD3/CD28 and cultured for 5 days. Secreted interleukins, cytokines and chemokines were measured in culture supernatants by multiplex ELISA.

Interleukin /Cytokine	CD4⁺ cells pg/ml (±SD) [1)]	CD8⁺ cells pg/ml (±SD) [1)]
IL-2	147500±23335	11900±283
INF-γ	20850±2475	44400±707
TNF-α	10625±1378	4370±509
IL-10	348±33	46±2
IL-13	1885±431	7035±1845
IL-5	198±42	572±163
IL-9	3805±574	620±187
IL-6	607±89	281±39
IL-17	300±15	44±0
CCL5/RANTES	8830±876	27850±502
CXCL8/IL-8	1365±149	1240±85
CXCL10/IP-10	5725±146	28050±3040
MCP-1/CCL2	19±5	128±5
MIP-1α/CCL3	6025±827	10990±1428
MIP-1β/CCL4	12250±1060	31550±406

[1)] N=3

Table S1b: Production of interleukins and cytokines by CD4$^+$ and CD8$^+$ subsets

CD4$^+$ or CD8$^+$ selected PBMC subsets were activated with anti-CD3/CD28 and cultured for 5 days. Secreted interleukins, cytokines and chemokines were measured in culture supernatants by multiplex ELISA.

Interleukin /Cytokine	CD4$^+$ cells pg/ml (±SD) [1)]	CD8$^+$ cells pg/ml (±SD) [1)]
IL-2	147500±23335	11900±283
INF-γ	20850±2475	44400±707
TNF-α	10625±1378	4370±509
IL-10	348±33	46±2
IL-13	1885±431	7035±1845
IL-5	198±42	572±163
IL-9	3805±574	620±187
IL-6	607±89	281±39
IL-17	300±15	44±0
CCL5/RANTES	8830±876	27850±502
CXCL8/IL-8	1365±149	1240±85
CXCL10/IP-10	5725±146	28050±3040
MCP-1/CCL2	19±5	128±5
MIP-1α/CCL3	6025±827	10990±1428
MIP-1β/CCL4	12250±1060	31550±406

[1)] N=3

Table S2: Prototypic T_h1 and T_h2 interleukins produced by anti-CD3/CD28 stimulated $CD4^+$ lymphocytes in the presence of vitamins and vitamin metabolites
Cells were cultured for 5 days and the secreted interleukins determined by multiplex ELISA

Retinoic Acid and VA

IL-2	Retinoic Acid [nM]	IL-2 [ng/mL]	Vitamin A [nM]	IL-2 [ng/mL]
	-	66.1 ± 2.5 [1)]	-	66.1 ± 2.5
	0.01	102.0 ± 3.3	10	103.7 ± 21.6
	0.10	118.1 ± 8.1	100	97.0 ± 2.5
	1.00	166.4 ± 8.5	1000	110.0 ± 11.4

1,25(OH)D3 and VD

IL-13	$1,25(OH)_2D_3$ [nM]	IL-13 [pg/mL]	Vitamin D3 [nM]	IL-13 pg/mL]
	-	1734 ± 341	-	1725± 177
	0.01	3401 ± 239	1	1945 ± 162
	0.10	5808 ± 598	10	2885 ± 374
	1.00	9789 ± 1045	100	4175 ± 7

[1)] Mean ± SD of triplicate cultures
